# Sociodemographic and socioeconomic correlates of learning disability in preterm children in the United States

**DOI:** 10.1186/s12889-022-12592-4

**Published:** 2022-02-01

**Authors:** Menkeoma Laura Okoli, Chukwuemeka E. Ogbu, Chioma O. Enyi, Ibuchim C. Okoli, Ronee E. Wilson, Russell S. Kirby

**Affiliations:** 1 Department of Epidemiology and Biostatistics, College of Public Health, University of South Florida, 13201 Bruce B Downs Blvd, Tampa, FL USA; 2 Menninger Department of Psychiatry and Behavioral Sciences, Baylor College of Medicine, Houston, TX USA; 3 Department of Clinical sciences, All Saints University (SVG), Arnos Vale, Saint Vincent and the Grenadines; 4 Department of Community and Family Health, College of Public Health, University of South Florida, Tampa, USA

**Keywords:** Learning disability, CLWLD, SES, Preterm birth, Special services

## Abstract

**Background:**

In 2019, 1 in every 10 infants born in the United States was preterm. Prematurity has life-threatening consequences and causes a range of developmental disabilities, of which learning disability is a prevalent complication. Despite the availability of special services for children living with learning disability, gaps still exist in terms of access due to socioeconomic factors. The aim of this study is to evaluate socioeconomic and sociodemographic correlates of learning disability in preterm children.

**Methods:**

This cross-sectional study used data from the 2016–2018 National Survey of Children’s Health. Weighted multivariable analyses were conducted to ascertain the association of sociodemographic and socioeconomic factors on learning disability among preterm children. The main outcome variable was the presence of learning disability.

**Results:**

Among 9555 preterm children in our study population, 1167 (12%) had learning disability. Learning disability was significantly associated with health insurance, food situation, and poverty level after adjustment for other variables. Children currently insured had lower odds of having learning disability compared to those without health insurance (OR = 0.79, 95% C.I. = 0.70–0.91). Also, children living in households that cannot afford nutritious meals are more likely to have learning disability compared to those that can afford nutritious meals at home (OR = 1.55, 95% C.I. = 1.22–1.97).

**Conclusion:**

These findings highlight the need for intervention efforts to target these children living with a learning disability to achieve the 2004 Individuals with Disabilities Education Act of promoting educational equality and empowerment of children living with a learning disability.

## Introduction

Preterm birth is defined as “the delivery of a baby before 37 completed weeks of pregnancy” [1, 2]. Globally, about 15 million preterm births are reported annually [3]. Current data suggest an increasing prevalence of preterm births in the United States given that in 2019, 1 in every 10 infants born was preterm [1, 2, 4, 5]. Preterm births in the United States presents a significant economic burden as more than $26 billion was spent in the management of cases in 2005 and this cost has more than tripled in recent times given increasing prevalence [2]. Prematurity at birth is associated with serious life-threatening conditions such as breathing difficulties, intracranial hemorrhage, developmental delay, all of which can result in neonatal mortality [1]. However, in cases where the child survives, a range of developmental disabilities are seen, of which learning disability is a prevalent form of complication [1, 2, 4].

Learning disability implies “having difficulty in one or more areas of learning even in the presence of normal intelligence” [6]. Globally, about 92 million children were found to be living with learning disability in 2011 [7]. Learning disability has numerous public health implications as studies have shown that children with this disability show poorer school outcomes compared to their normal peers [2, 8, 9]. Studies have shown the rate of placement of children with learning disability in special services programs to have doubled over time with these programs constituting a significant economic burden as they cost more than triple of general education services [8]. In 2019, more than 2 million children in the United States received special services due to learning disabilities [10].

Both medical and behavioral approaches in the management of children living with learning disability (CLWLD) have in most cases proven beneficial in improving the quality of life of CLWLD, however, it is also important to consider the significant role sociodemographic and socioeconomic factors play on health outcomes in these children [8, 11]. Therefore, examining sociodemographic and socioeconomic factors affecting these CLWLD offers another approach to tackling the issue. Studies have shown that sociodemographic and socioeconomic variables such as maternal education, poverty, and race have effects on the educational outcomes of children and these effects are even more pronounced as the children go through their educational program [9, 12, 13].

Children from families with higher socioeconomic status (SES) living with learning disability are more likely to get into integrated special services programs tailored to their special needs compared to those from families with lower SES [8, 14–17]. Additional studies have also associated children from lower SES with learning disabilities to have a higher probability of repetition of grades, and in other instances, dropping out of high school, thereby amounting to reduced economic workforce [9, 18–20].

Few studies have examined sociodemographic factors and their relationship to learning disability in individual states in the United States [8, 18, 21]. To the best of my knowledge, no study has explored the relationship of socioeconomic and sociodemographic factors and learning disability in preterm children for all the states combined. Providing evidence of association of socioeconomic and sociodemographic factors with learning disability in preterm children can offer other methods to address this issue aside from the currently available clinical and behavioral treatment modalities and can open discussion on working to address these factors.

Therefore, the aim of this study is to evaluate the relationship between socioeconomic and sociodemographic factors and learning disability in preterm children aged 3–17 years in the United States.

## Methods

### Study design and data source

The National Survey of Children’s Health (NSCH) dataset (2016–2018) [22] was used for this analysis. The NSCH is a representative sample survey that offers state and national estimates concerning the health and well-being of United States children aged 0–17 years. Parents/caregivers of children between the ages of 0–17 years in different households all over the United States were survey respondents. Questionnaire administration and  data collection were carried out by the United States Census Bureau. Survey questionnaires collected data on demographics, health insurance coverage, health indicators, health care utilization, and access, childhood health issues, family health status and functioning, and neighborhood characteristics. The response rate in the 3-year period ranged from 37 to 43%. The same sampling design was utilized for the three years (2016–2018) combined dataset. A thorough description of the data sampling methods and other analytical guidelines have been described elsewhere [22, 23].

### Study population

The sample population included 102,341 children between the ages of 0–17 years. This analysis was restricted to children between the ages of 3–17 years who were born preterm (*n* = 9591). Records with missing data on study variables were excluded (*n* = 36). The final study population consisted of 9555 preterm children aged 3–17 years. This age group (children between the ages of 3–17 years) was selected as studies have shown the clinical signs of learning disability to be more apparent from three years and above [1, 4].

### Study variables

The outcome variable for this analysis was the presence of learning disability as reported by a parent for a child as indicated by a physician’s diagnosis and classified as presence or absence (Table 1). The independent variables include sociodemographic factors such as sex, categorized as female or male, race/ethnicity categorized as Hispanic, White non-Hispanic, Black non-Hispanic and multiracial, educational level of child’s parent or guardian was grouped as less than high school or high school degree and some college or higher education, family structure grouped as two parents, single parents, grandparent or other family types, age grouped as (3–5 years old- Preschoolers, 6–13 years old- Middle childhood, and 14–17 years old- Teenagers). Socioeconomic factors included in the analysis are Federal poverty level (FPL), grouped as 0–99% FPL, 100–199% FPL, 200–399% FPL, 400% FPL or above, health insurance categorized as currently and adequately insured, not currently and adequately insured, and food situation at home classified as can afford nutritious meals, cannot afford nutritious meals). All variables used in this study are similar across the years (2016–2018) and are measured in the same manner across the 3-year period.

**Table 1:** Characteristics of Learning disability in preterm children by selected sociodemographic and socioeconomic variables (NSCH 2016–2018)

Variables	Total	Presence	Absence	*P*-value
	(*n* = 9555)	(*n* = 1167)	(*n* = 8388)	
	N (%)	N (%)	N (%)	
Race				<.0001
White Non-Hispanic	6540 (68.45)	774 (66.32)	5766 (68.74)	
Hispanics	1139 (11.92)	141 (12.08)	998 (11.90)	
Black Non-Hispanic	704 (7.37)	128 (10.97)	576 (6.87)	
Multiracial	1172 (12.27)	124 (10.63)	1048 (12.49)	
Sex				<.0001
Male	5152 (53.92)	744 (63.75)	4408 (52.55)	
Female	4403 (46.08)	423 (36.25)	3980 (47.45)	
Health Insurance				0.0008
Currently and adequately insured	3306 (34.68)	454 (39.07)	2852 (34.07)	
Not currently and adequately insured	6226 (65.32)	708 (60.93)	5518 (65.93)	
Poverty level				<.0001
0–99%FPL	1187 (12.42)	226 (19.37)	961 (11.46)	
100–199%FPL	1636 (17.12)	270 (23.14)	1366 (16.29)	
200–399%FPL	2949 (30.86)	337 (28.88)	2612 (31.14)	
400% FPL and above	3783 (39.59)	334 (28.62)	3449 (41.12)	
Family structure				<.0001
Two parents	7192 (76.38)	770 (67.25)	6422 (77.64)	
Single parent	1711 (18.17)	268 (23.41)	1443 (17.45)	
Other Family type/Grandparent	513 (5.45)	107 (9.34)	406 (4.91)	
Adult educational level				<.0001
High school or less	1582 (16.75)	264 (23.08)	1318 (15.87)	
Some College or higher	7865 (83.25)	880 (76.92)	6985 (84.13)	
Food situation at home				<.0001
Cannot afford nutritious meals	487 (5.21)	108 (9.54)	379 (4.61)	
Can afford nutritious meals	8869 (94.79)	1024 (90.46)	7845 (95.39)	
Age				<.0001
3–5 years	1563 (16.36)	81 (6.94)	1482 (17.67)	
6–13-years	4827 (50.52)	631 (54.07)	4196 (50.02)	
14–17 years	3165 (33.12)	455 (38.99)	2710 (32.31)	

Counts and percentages represent unweighted frequencies and weighted percentages of study population. *P*-value is derived from the Rao-Scott Chi-Square test

### Ethical approval

The Maternal and Child Health Bureau (MCHB) received approval from the National Center for Health Statistics Research Ethics Review Board, and all participants provided informed consent. This study is exempt from full institutional review board review because the NSCH is a publicly available dataset.

### Statistical analysis

Given the complex sample survey design, procedures to properly account for the survey methods were used in the analysis, with weighted multivariable analyses. Study population characteristics were examined using unweighted frequencies and weighted percentages. Differences in proportions of socioeconomic factors among children with and without learning disability were examined using Rao-Scott Chi-square for complex survey design. Crude odds ratios were obtained by analyzing each variable with the outcome variable-presence of learning disability using weighted logistic regression models. All socioeconomic and sociodemographic variables: health insurance, food situation at home, federal poverty level, educational level, family structure, race, educational level, and sex were included in a weighted multiple logistic regression model to determine the variables independently associated with the presence of learning disability. Given the documented possibility of interaction between educational level and health insurance by learning disability status, [24] we tested for interaction in another model. Odds ratios and their corresponding 95% confidence intervals were subsequently reported. Data analysis was conducted using SAS version 9.4 (Cary, NC).

## Results

The study population comprised 9555 preterm children aged 3–17 years. From this population, 1167 (12%) had a parent or caregiver who reported a physician diagnosis of learning disability (Table 1). About 12% of the study population was classified in the federal poverty level category of 0–99% FPL. Approximately 76% of households had a  two-parent structure. More than half of children were not currently and adequately insured (65%). Most children were White, non-Hispanic (68.5%), followed by multiracial group (12.3%), and Black, non-Hispanic (7.4%). About 11.9% of children were of Hispanic race. About 53.9% of these children were of male gender whereas 46.1% were female.

With respect to the educational level of parents or caregivers, 16.8% attained less than high school or high school education, 83.3% reported some college education or higher. In terms of food situation at home, 94.8% reported being able to afford nutritious meals whereas 5.2% of these households could not afford nutritious meals. Most of the children were in the 6–13 years age group (50.5%), and 33.1% were in the 14–17 years age group and 16.4% of these children were in the 3–5 years age group.

Table 1 illustrates the distribution of socioeconomic and sociodemographic characteristics across the study population. Statistically significant differences were found in the proportion of these characteristics. Among CLWLD, 39.1% of children were found to be currently and adequately insured, compared to 34.1% of those without learning disability, similarly, 61% of CLWLD have no current or adequate health insurance compared to 66% of children without learning disability (*p* = 0.0008). About 41.1% of families of children without learning disability were found to be within the 400% federal poverty level compared to 28.6% of CLWLD (*p* = <.0001). For CLWLD, 63.8% are of male gender compared to 52.6% without learning disability (*p* = <.0001).

Approximately 76.9% of parents of CLWLD had a college or more education compared to 84.1% of parents of children without learning disability (*p* = <.0001). Among CLWLD, 67.3% are from a two-parent family structure, compared to 77.6% of those without learning disability (*p* = <.0001). For CLWLD, approximately 90.5% can afford nutritious meals compared to 95.4% of those without learning disability (*p* = <.0001). About 32.3% of children without learning disability are in the age category of 14–17 years, compared to 39% of CLWLD (*p* = <.0001). The majority of CLWLD (66.3%) are White non-Hispanic compared to 68.7% of children that do not have learning disability (*p* = <.0001).

Table 2 below shows the crude and adjusted odd ratios (OR) of the association between socioeconomic/ sociodemographic factors and the presence of learning disability in children. Health insurance, food situation at home, federal poverty level, and sex remained as independent predictors of the presence of learning disability in the adjusted model (model 1).

**Table 2:** Regression analysis for the association of sociodemographic and socioeconomic factors and learning disability among preterm children aged 3–17 years old (NSCH 2016–2018)

Variables	Crude Odd Ratios (95% CI)	Model 1^a^ (95% CI)	Model 2^b^ (95%) CI
Race			
Hispanics	1.05 (0.87–1.28)	0.91 (0.74–1.12)	0.92 (0.75–1.12)
Black Non-Hispanic	1.66 (1.35–2.03)	1.33 (1.06–1.66)	1.33 (1.06–1.67)
Multiracial	0.88 (0.72–1.08)	0.83 (0.67–1.03)	0.83 (0.67–1.03)
White Non-Hispanic	Reference	Reference	Reference
Sex			
Male	1.59 (1.40–1.80)	1.65 (1.44–1.88)	1.65 (1.44–1.88)
Female	Reference	Reference	Reference
Health Insurance			
Currently and adequately insured	0.81 (0.71–0.91)	0.79 (0.70–0.91)	
Not currently and adequately insured	Reference	Reference	
Poverty level			
0–99%FPL	2.43 (2.02–2.92)	1.87 (1.50–2.34)	1.85 (1.48–2.31)
100–199%FPL	2.04 (1.72–2.43)	1.80 (1.46–2.14)	1.76 (1.55–2.14)
200–399%FPL	1.33 (1.14–1.56)	1.26 (1.10–1.49)	1.26 (1.07–1.49)
400% FPL and above	Reference	Reference	Reference
Family structure			
Single parent	1.55 (1.33–1.80)	1.16 (1.00–1.37)	1.15 (0.97–1.36)
Other Family type/Grandparent	2.20 (1.75–2.76)	1.80 (1.40–2.30)	1.80 (1.41–2.31)
Two parents	Reference	Reference	Reference
Adult educational level			
High school or less	1.59 (1.37–1.85)	1.15 (0.97–1.36)	
Some College or higher	Reference	Reference	
Food situation at home			
Cannot afford nutritious meals	2.18 (1.75–2.73)	1.55 (1.22–1.97)	0.65 (0.51–0.82)
Can afford nutritious meals	Reference	Reference	Reference
Age			
3–5-year-old	0.33 (0.26–0.42)	0.33 (0.26–0.43)	2.74 (2.14–3.49)
6–13-year-old	0.90 (0.79–1.02)	0.91 (0.80–1.05)	3.00 (2.33–3.86)
14–17-year-old	Reference	Reference	Reference
Adult education *Insurance			
Currently and adequately insured (High school or less vs Some college or higher)			0.79 (0.59–1.06)
Not Currently and adequately insured (High school or less vs Some college or higher)			1.39 (1.14–1.70)

*CI* Confidence Interval

^a^Model adjusted for age, sex, race, adult education, family structure, food situation at home, health insurance, federal poverty level

^b^Interaction model: adjusted for age, sex, race, adult education, family structure, food situation at home, health insurance, federal poverty level

In the bivariate analysis, children who are currently and adequately insured had 20% lower odds of having learning disability compared to those without adequate health insurance (OR = 0.81, 95% C.I. = 0.71–0.91). This effect was persistent after controlling for other variables in the multivariate analysis (OR = 0.79, 95% C.I. = 0.70–0.91). Similarly, in the bivariate analysis, children living in households that cannot afford nutritious meals are 2.18 times more likely to have learning disability compared to families of children that can afford nutritious meals at home (OR = 2.18, 95% C.I. = 1.75–2.73). This association persisted after adjustment for other variables in model 1 (OR = 1.55, 95% C.I. = 1.22–1.97).

Additionally, families in the lowest income level (0–99% FPL) are 2.43 times more likely to have children with learning disability compared to those in the highest income level (400% FPL) (OR = 2.43, 95% C.I. = 2.02–2.92). This association remained consistent after controlling for other variables in model 1 (OR = 1.87, 95% C.I. = 1.50–2.34). Also, in the bivariate analyses, male children had greater odds of having learning disability compared to the female children (OR = 1.59, 95% C. I = 1.40–1.80), this association remained after controlling for other variables (OR = 1.65, 95% C. I = 1.44–1.88).

Educational level, although significant in the crude bivariate analysis, was not significant in the multivariable  analysis. Parents or caregivers with less than high school and high school education, were 1.59 times more likely to have children with learning disability compared to those with some college degree or higher (OR = 1.59 95% C.I. = 1.37–1.85).

Given the documented possibility of interaction between educational level and health insurance by learning disability status, [24] we tested for interaction in another model, model 2 (Table 2) and it was significant. The association between parents’ level of education and learning disability in children differed by insurance status. In children who do not have current and adequate insurance, the odds ratio of parent’s educational status (high school and less vs. some college and higher) and learning disability was higher (OR = 1.39 95% C. I = 1.14–1.70) compared to the odds ratio of the same association in parents who had current and adequate insurance (OR = 0.79 95% C. I = 0.59–1.06).

## Discussion

The aim of this study was to evaluate the relationship between sociodemographic/socioeconomic factors and learning disability in preterm children. Findings from this study showed health insurance, federal poverty level, food situation at home, and sex to be significantly associated with learning disability in children. Children who are not currently and adequately insured had greater odds of having learning disability compared to those with current and adequate health insurance. This finding is consistent with the literature as studies have shown extremely low health care utilization in children with special needs, despite being the category that needs healthcare the most, given associated health issues [7, 25–28]. This finding highlights a gap in health care access for families with children living with learning disabilities (CLWLD) in terms of obtaining health care consultation for their children as well as obtaining coverage for necessary medications, potentially placing CLWLD at risk of severe health complications.

Previous studies have documented an increasing amount of emergency care visits and hospitalizations for children with special care needs due to a lack of adequate health insurance [27, 28]. Although several health insurance plans are available for children under the Affordable Care Act, especially for families of low-income including Medicaid, Children’s Health Insurance Program (CHIP), and qualified health plans (QHPs) [ 27, 28], however, several issues such as cost-sharing between families and insurance companies as well as high out of pocket costs still present as limiting factors for health care utilization [27, 28].

Findings from this study also showed that children living in households that cannot afford nutritious meals were more likely to have learning disability compared to children living in households that can afford nutritious meals. From a clinical perspective, proper and adequate nutrition is necessary for neurodevelopment, body growth, and functioning of the organ system. Similar findings have been reported in other studies as poor nutrition has been shown to cause less participation in school activities, poor concentration during classes, tardiness, and in some cases can result in socially unacceptable behaviors like stealing from peers [29]. Such action can cause this group of children to be treated differently and isolated from their peers, giving rise to issues such as low self-esteem, anxiety, aggression, and stress, all of which can severely affect child learning and behavior [ 27, 29]. Additional studies have linked poor nutrition in childhood to the development of permanent structural brain damage along with the development of chronic illness such as obesity, heart diseases, and diabetes, later in life [27, 29, 30].

Another important finding is that families of children in the lowest income level were more likely to have children with learning disability compared to those in the highest income level. Data from previous studies show that in 2013, about 40% of CLWLD in the United States were found to live in households within the lowest income category of 100–200% FPL [26, 27]. Additional studies have highlighted the restrictive effect of poverty in the ability of families to access special services for their CLWLD, [26, 31, 32] along with differences in terms of race or ethnicity, as African American or Black CLWLD were less likely to access special services in relation to their White peers [22, 27]. This disproportionality cuts across socioeconomic factors and the availability of resources for minority populations. The importance of this finding can be viewed as poverty being the root cause of subsequent socioeconomic effects affecting CLWLD, as it directly impacts both food situation at home as well as access to adequate health insurance.

While substantial strides have been made by the government in addressing the issue of poverty especially for low-income families, in the manner of provision of food stamps, cash or voucher programs- supplement security income [26], a lot more still needs to be done given prevailing conditions of families with CLWLD in the low-income category.

Consistent with other studies where gender differences have been shown to exist with respect to learning disability cases [7, 21], our study found that male children had a higher likelihood of having learning disability compared to females. The etiology of these differences remains unclear [21].

In model 2, findings from the interaction analysis between adult education and health insurance for CLWLD weres statistically significant. Similar interaction analysis has been examined in a previous study [24]. Learning disability was associated with educational status in the uninsured but was not significant in the insured group. Compared to parents with some college or higher, parents with less than high school or high school education who were uninsured had a higher odds of learning disability in their children. This finding underscores the protective effect of health insurance in the association between learning disability and parental education, suggesting that the presence or absence of health insurance could be a major moderator of socioeconomic factors like parental educational status on outcomes like learning disability, although more longitudinal analyses are needed to clarify this effect. Given the critical state of health insurance in the United States, as the U. S Census Bureau’s Health insurance coverage report stated that in 2018, more than 4 million children had no health insurance coverage [33], it is highly imperative that attention is placed on CLWLD without current and adequate health insurance.

The presence of a learning disability in a child is associated with numerous health and social consequences including repetition of grades, dropping out of high school, peer rejection, anxiety, as well as behavioral and conduct problems [ 16–18, 34, 35]. In addition, a study has shown a particular form of learning disability in children, nonverbal type to be associated with depression and suicide [36]. Additionally, families of CLWLD experience  various range of effects such as poor mental health due to associated stress, marital issues, interruption of work and career plans [26, 29, 30, 37], and on their siblings can cause relationship and behavioral issues as they perceive lack of attention from parents [38, 39]. Given the consequences associated with learning disability irrespective of socioeconomic status, the magnitude of these consequences arise in a dose-response fashion for families in the low socioeconomic category with increasing stress on the family which ultimately affects CLWLD in such families.

The future expectations of most youths includes graduating from school, getting a job, and being financially dependent irrespective of disabilities, however, CLWLD tends to have slower progress in achieving these goals, and this progress is increasingly impeded for CLWLD from families with low socioeconomic status [40]. Therefore, efforts aimed at addressing the root causes of learning disability, particularly socioeconomic factors, in order to close the gaps in accessing special services for CLWLD as well as providing resources for families in managing CLWLD would prove invaluable in mitigating these adverse health and social consequences as well as protect and promote the health and future success of children with disabilities.

Findings from this study highlights the important role sociodemographic and socioeconomic factors play as determinants of progression of learning disability in preterm children and provide an avenue for solutions to be developed to address these factors. Possible recommendations for tackling this issue will involve collaboration between public health professionals, health care providers, community leaders, government, families, special services, and education programs school staff team and includes: Promoting school’s commitment to active communication with families of CLWLD to foster proper understanding of demands, opportunities as it relates to their children’s educational needs, provision of healthier and more nutritious food options at home and in schools, development, and implementation of policies by the government that addresses the imbalance in access to special services for CLWLD, developing community-based supports groups to help engage families with CLWLD to enable them to cope with challenges and stress as well as providing mental health services and support to families with CLWLD [41–43].

Also, intervention efforts targeting parental education, especially maternal education will be instrumental in effecting a more lasting impact on the prevalence of preterm births and subsequently, learning disability. Efforts aimed at addressing these socioeconomic effects will help promote child education, engagement, and student success in line with the 2004 Individuals with Disabilities Education Act (IDEA) that advocates for the educational advancement of children living with disabilities to prepare them for future opportunities [44].

Data from the National Survey of Children’s Health (NSCH), a nationally representative data source, was used for this study. Various limitations exist for this study and include the cross-sectional nature of the study, therefore making it difficult to ascertain the direction of causality. Furthermore, the data collected was based on self-report and could be prone to recall and social desirability bias. In addition, certain putative confounders like genetic diseases that predispose a child to learning disabilities were not controlled for in this analysis due to unavailability in the  dataset. Given the large variation in outcomes for preterm children according to gestational age, it would have been important to include gestational age in the model, however, this was not available in the dataset. While this current study focused on sociodemographic and socioeconomic factors, future studies exploring social relationships such as teacher to student, peer to peer, as it relates to learning disability as well as maternal mental health as it relates to birth outcomes and child-rearing might be warranted to gain deeper insight in improving the health outcomes of children with learning disabilities.

## Conclusion

These findings illustrate the importance of government policies that address the inaccessibility of special services programs for minority CLWLD, parental or maternal health education, provision of mental health support services for families with CLWLD, and school and community engagement of families with CLWLD, to help mitigate the gap from social inequities, which are all necessary  for achieving the 2004 Individuals with Disabilities Education Act of promoting child educational equality and empowerment.

## Data Availability

The datasets generated and/or analysed during the current study are available in the repository- Data Resource Center for Child and Adolescent Health (https://www.childhealthdata.org/) [22]

## Abbreviations

CLWLD: Children living with learning disability
FPL: Federal Poverty level
